# *Spirocamallanus rarus* (Nematoda: Camallanidae) in Brazil: a taxonomic revision and new host record

**DOI:** 10.1590/S1984-29612026024

**Published:** 2026-07-20

**Authors:** Raul Henrique da Silva Pinheiro, Patrick José Colares Cardoso, Tiago Paixão Mangas, Michelle Cristie Gonçalves da Fonseca, Luis Augusto Araújo dos Santos Ruffeil, Marcelo Knoff, Elane Guerreiro Giese

**Affiliations:** 1 Universidade Federal Rural da Amazônia – UFRA, Instituto da Saúde e Produção Animal – ISPA, Laboratório de Histologia e Embriologia Animal – LHEA, Belém, PA, Brasil; 2 Universidade Federal Rural da Amazônia – UFRA, Instituto da Saúde e Produção Animal – ISPA, Programa de Pós-graduação em Saúde e Produção Animal na Amazônia – PPG-SPAA, Belém, PA, Brasil; 3 Instituto Federal de Educação, Ciência e Tecnologia do Pará – IFPA, Breves, PA, Brasil; 4 Fundação Oswaldo Cruz – FIOCRUZ, Instituto Oswaldo Cruz, Laboratório de Helmintos Parasitos de Vertebrados, Rio de Janeiro, RJ, Brasil; 5 Escola Estadual de Ensino Técnico do Pará Doutor Celso Malcher, Belém, PA, Brasil

**Keywords:** Camallanidae, Spirocamallanus rarus, taxonomic revision, Propimelodus eigenmanni, Brazilian Amazon, Camallanidae, Spirocamallanus rarus, revisão taxonômica, Propimelodus eigenmanni, Amazônia brasileira

## Abstract

*Spirocamallanus rarus* (Travassos, Artigas & Pereira, 1928) is reported for the first time parasitizing the intestine of *Propimelodus eigenmanni* (Van der Stigchel, 1946) collected from the Parauaú River, in Breves municipality, Pará, Brazil. Using light and scanning electron microscopy, several morphological aspects of this species were clarified and expanded upon. Newly described features include details of the cephalic structures, cuticular topography, the number and distribution of the caudal papillae in males, and the morphology of the vulva, anus and the phasmids on the tail in females. In this study, *S*. *rarus* was redescribed based on these new observations and a supplemental morphological re-study of the holotype and voucher specimens deposited in the Helminthological Collection of the Oswaldo Cruz Institute.

## Resumo

*Spirocamallanus rarus* (Travassos, Artigas & Pereira, 1928) é relatado pela primeira vez como parasito do intestino de *Propimelodus eigenmanni* (Van der Stigchel, 1946), coletado no rio Parauaú, no município de Breves, Pará, Brasil. Utilizando microscopia de luz e eletrônica de varredura, vários aspectos morfológicos desta espécie foram esclarecidos e ampliados. As características recém-descritas incluem detalhes das estruturas cefálicas, topografia cuticular, número e distribuição das papilas caudais nos machos e morfologia da vulva, ânus e fasmídeos na cauda nas fêmeas. No presente estudo, *S. rarus* foi redescrito com base nessas novas observações e num reestudo morfológico suplementar do holótipo e dos espécimes de referência depositados na Coleção Helmintológica do Instituto Oswaldo Cruz.

## Introduction

*Propimelodus eigenmanni* (Van der Stigchel, 1946) belongs to the family Pimelodidae, commonly known as the long-whiskered catfishes (order Siluriformes). *Propimelodus eigenmanni* has been been reported as occurring in the Amazon, Approuague, Kourou, and Oyapock River basins (Froese & Pauly, 2025).

Camallanidae Railliet & Henry, 1915 is a monophyletic family of nematodes, species of which infect fish in the most varied aquatic environments worldwide (Anderson et al., 2009; Černotíková et al., 2011). The majority of studies on the taxonomy of Camallanidae have been based on the morphology of their structures, especially in relation to the species of *Procamallanus* Baylis, 1923, given that few species have been genetically characterized, and phylogenetic relationships are poorly understood (Wu et al., 2008; Kuzmin et al., 2011; Gaither et al., 2013; Sardella et al., 2017).

Classification of the genera within the Camallanidae is usually based on the morphology of the buccal capsule (Anderson et al., 2009; Rigby & Rigby, 2014). Authors disagree on how decisive the taxonomic weight of this characteristic is in differentiating specimens at the genus or subgenus level, and this has generated considerable debate on the subject. The genus *Procamallanus* is subdivided into five subgenera; specimens with a buccal capsule possessing chitinoid bands arranged in spiral are classified within the subgenus *Spirocamallanus* (Moravec & Sey, 1988; Moravec & Thatcher, 1997; Rigby & Adamson, 1997; Moravec, 1998; Anderson et al., 2009; Moravec & Van As, 2015a, b). Other authors treat this as a distinct genus (Andrade-Salas et al., 1994; Rigby & Rigby, 2014; Ramallo & Ailán-Choke, 2017; Pinheiro et al., 2018; Svitin et al., 2019; Santos Reis et al., 2021). With the advance of molecular analyses, some authors have demonstrated that the generic classification considering *Spirocamallanus* as genus should be adopted (Rigby & Rigby, 2014; Ailán-Choke & Pereira, 2021).

Various species of *Spirocamallanus* have been reported in Brazil parasitizing fish of different orders in marine, freshwater and estuarine environments, namely, as listed below, *S. inopinatus* (Travassos, Artigas & Pereira, 1928); *S. iheringi* (Travassos, Artigas & Pereira, 1928); *S. rarus* (Travassos, Artigas & Pereira, 1928); *S. amarali* (Vaz & Pereira, 1934); *S. hilarii* (Vaz & Pereira, 1934) (=*S. cearensi* and *S. incarocai*); *S. barroslimai* (Pereira, 1935); *S. pereirai* (Annereaux, 1946); *S. macaensis* (Vicente & Santos, 1972); *S. pimelodus* (Pinto, Fabio, Noronha & Rolas, 1974) (= *S. intermedius*); *S. solani* (Pinto, Fabio, Noronha & Rolas, 1975); *S. paraensei* (Pinto & Noronha, 1976); *S. pexatus* (Pinto, Fabio, Noronha & Rolas, 1976); *S. cruzi* Guimarães, Cristofaro & Rodrigues, 1976; *S. caballeroi* Bashirullah, 1977; *S. halithophus* Fusco & Overstreet, 1978; *S. pintoi* Kohn & Fernandes, 1988; *S. freitasi* Moreira, Oliveira & Costa, 1991; *S. saofranciscencis* Moreira, Oliveira & Costa, 1994; *S. belenensis* Giese, Santos & Lanfredi, 2009, *Spirocamallanus krameri* Petter, 1974 (Petter, 1979; Luque et al., 2011; Eiras et al., 2016; Pinheiro et al., 2020; Santos Reis et al., 2021). Recently, an additional species has been described, *S. delirae* Ruffeil, Giese & Pinheiro, 2023 from *P. eigenmanni* catfish, collected in the Breves municipality, Pará state (Ruffeil et al., 2023).

As part of an ongoing study of camallanid nematodes in fish from the Brazilian Amazon, samples of *P. eigenmanni* were collected and necropsied. Procamallaninae Yeh, 1960 nematodes were recovered from the intestines of these fish and were attributed to *S*. *rarus*. This nematode species has been reported occurring throughout much of the Neotropical region in freshwater fishes, from Brazil, Paraguay, Argentina, and Peru (Travassos et al., 1928; Travassos & Kohn, 1965; Pinto et al., 1974; Vicente et al., 1985; Kohn & Fernandes, 1987; Rodrigues et al., 1991; Moravec, 1998; Vicente & Pinto, 1999; Thatcher, 2006; Giese et al., 2009; Melo et al., 2012; Eiras et al., 2016; Santos Reis et al., 2021).

The aims of this study were to report the occurrence of the species *S. rarus* parasitizing *P. eigenmanni*, redescribe its morphology using optical and scanning electron microscopies, calculate the parasitic indices, and add new data on the biodiversity of camallanid nematodes parasitizing freshwater siluriform catfish in the Neotropical region.

## Material and Methods

Twenty-five specimens of *P. eigenmanni* catfish (15 females and 10 males), measuring 8–14 (11) cm standard length, 7–34 (17) g weight, were caught by fishers from the Parauaú River, Breves municipality (1°40'57''S, 50°28'51''W), Pará state in the eastern Brazilian Amazon. Fish were collected from March to July 2020 with a casting net. The “Instituto Chico Mendes de Conservação da Biodiversidade” authorized all procedures for collecting fish (SISBIO, N° 68028-2: Authentication code: 0680280220200412). The fish were identified according to Lundberg & Parisi (2002), Lundberg & Littmann (2003) and Froese & Pauly (2025). The fish collected were transported dead in coolers filled with ice to the laboratory for necropsy. In the laboratory, the organs were separated and placed in Petri dishes with 0.65% NaCl solution and examined using a stereomicroscope. Live nematodes collected from the intestines were fixed with hot AFA at 60°C, preserved in 70% ethanol 5% glycerin, clarified with Amman's lactophenol and examined using an Olympus BX–41 optical microscope. Illustrations were made using a drawing tube connected to the microscope. Some samples were analyzed by optical microscopy using a Zeiss Axiophot microscope equipped with a Nomarski differential interference contrast apparatus (DIC), and the images were obtained using a digital Leica DM2500 microscope with Leica DFC310 FX camera system with Leica Application Suite Software V4.4 (Leica Microsystems GmbH, Wetzlar, Germany). Measurements are provided in micrometers (µm) unless otherwise indicated, with range in parentheses. Scanning electron microscopy (SEM) was used to elucidate topographic details of specimens of *Spirocamallanus*. For scanning electron microscopy (SEM), helminths were washed in phosphate-buffered saline with a pH of 7.0 (Sodium Phosphate Monobasic 3.12 g, Sodium Phosphate Dibasic 2.83 g, and 17 g Sodium Chloride in 200 ml of distilled water), post-fixed in 1% osmium tetroxide, dehydrated to the critical point using CO2, coated in gold+palladium and examined and photographed using VEGA 3 LMU/TESCAN scanning electron microscope. Nematodes were processing according to Pinheiro et al. (2018, 2020). The taxonomic classifications of the nematodes were carried out according to De Ley & Blaxter (2002), and the species identification according to Travassos et al. (1928), Pinto et al. (1974) and Melo et al. (2012). Prevalence, mean intensity and mean abundance were calculated according to Bush et al. (1997). Range of infection and sites of infection are also presented. Representative specimens of the nematode species collected were deposited in the Coleção de Invertebrados Não Arthropoda of the Museu Paraense Emílio Goeldi (MPEG), Belém, PA, Brazil. The holotype and representative specimens used on the original description of the *S. rarus* (Travassos et al., 1928) and subsequent redescriptions (Pinto et al., 1974; Melo et al., 2012) deposited at CHIOC had their morphological characteristics reevaluated using an Olympus BX–41 and Zeiss Axiophot optical microscopes. The specimen collected by Melo et al. (2012), which was preserved as wet material (70% ethanol, 5% glycerin), was mounted in temporary preparation in Amman's lactophenol.

## Results

A total of 56 nematodes (31 females and 25 males) were collected from the intestines of nine *P. eigenmanni*. The species taxonomic identification follow.

Nematoda Pottis, 1932, Chromadorea Inglis, 1893, Chromadoria Pearse, 1942, Rhabditida Chitwood, 1933, Spirurina Railliet & Henry, 1915, Spiruromorpha De Ley & Blaxter, 2002, Camallanoidea Railliet & Henry, 1915, Camallanidae Railliet & Henry, 1915, *Spirocamallanus* Olsen, 1952

### *Spirocamallanus rarus* (Travassos, Artigas & Pereira, 1928) (Figures 1 [Fig gf02] [Fig gf03]-4)

**Figure 1:**
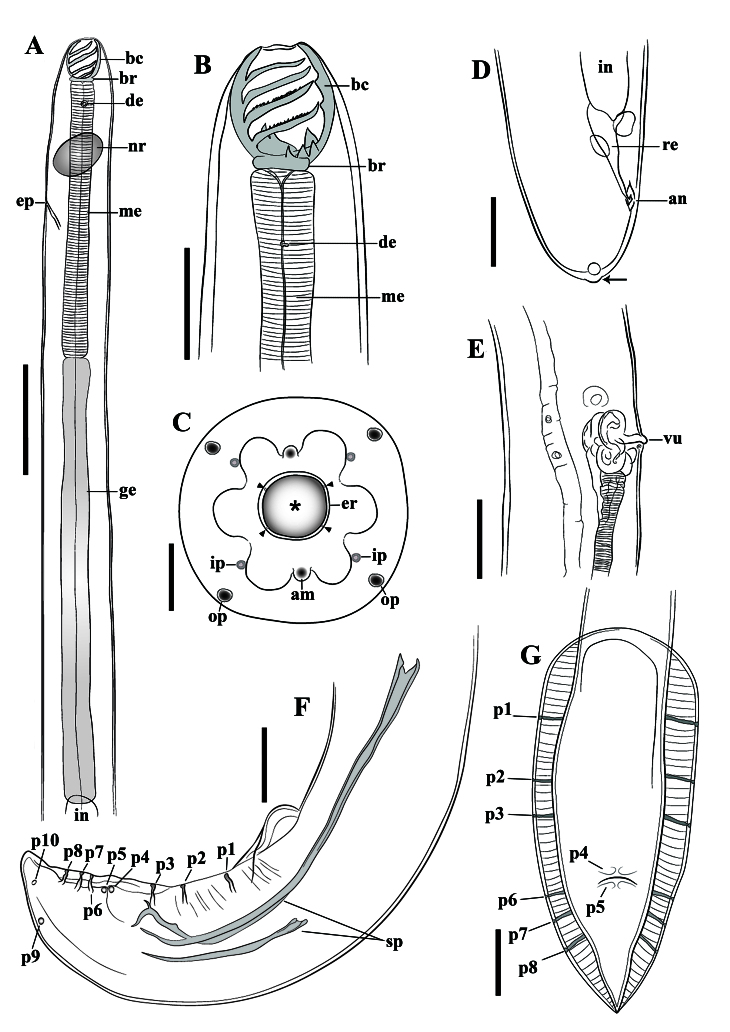
*Spirocamallanus rarus*parasitizing*Propimelodus eigenmanni*. (A) Male, anterior end, lateral view, showing buccal capsule (bc), basal ring (br), deirid (de), muscular esophagus (me), glandular esophagus (ge), nerve ring (nr), excretory pore (ep), and intestine (in). (B) Male, detail of cephalic region, lateral view, showing buccal capsule (bc), basal ring (br), teeth visible at the base of buccal capsule, deirid (de) and muscular esophagus (me). (C) Female, cephalic region, *en face* view, showing mouth opening (asterisks), with a thick cuticular external ring (er) at the anterior end of the buccal capsule, surrounded by inner circle with four small pore (arrowheads) at base proximal to mouth opening, six elevations, one ventral, one dorsal, and four lateral, and eight papillae distributed in two circles, four internal smaller (ip) and four outer larger (op) and a pair of small lateral amphids (am). (D) Female, tail, lateral view, showing intestine (in), rectum (re), anus (an), and the tail tip with the button-shaped cuticular projection (arrow). (E) Female, detail of vulvar region, lateral view, showing vulva (vu). (F) Male, posterior end, lateral view, showing caudal papillae, p1–p3 precloacal, p4–p5 adcloacal, p6–p10 postcloacal pairs (p9–p10 the phasmids), and spicules (sp). (G) Male, posterior end, ventral view, showing caudal papillae (p1–p3 precloacal, p4–p5 adcloacal and p6–p8 postcloacal pairs). Scale bars A, B, E = 100μm; C = 20 μm; D, F, G = 50 μm.

**Figure 2 gf02:**
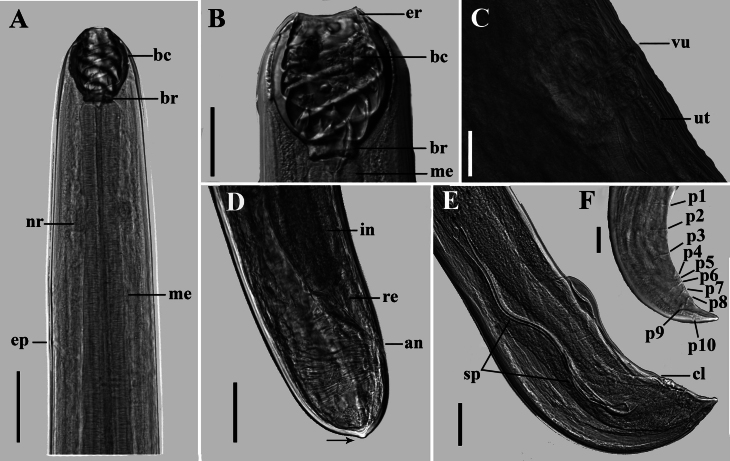
*Spirocamallanus rarus*parasitizing*Propimelodus eigenmann* by DIC. (A**)** Male, anterior end, lateral view, showing buccal capsule (bc), basal ring (br), muscular esophagus (me), nerve ring (nr) and excretory pore (ep). (B) Female, detail of buccal capsule (bc), lateral view, showing cuticular external ring (er), teeth at the base of the buccal capsule, basal ring (br) and muscular esophagus (me). (C) Female, detail of vulvar region, lateral view, showing vulva (vu) and uterus (ut). (D) Female, tail, lateral view, showing intestine (in), rectum (re), anus (an), and the tail tip with button-shaped cuticular projection (arrow). (E–F) Male posterior end, lateral view, showing spicules (sp) and cloacal opening (cl), caudal papillae, p1–p3 precloacal, p4–p5 adcloacal, p6–p10 postcloacal pairs (p9–p10 the phasmids). Scale bars: A, B, D and E = 100 μm, C and F = 50 μm.

**Figure 3 gf03:**
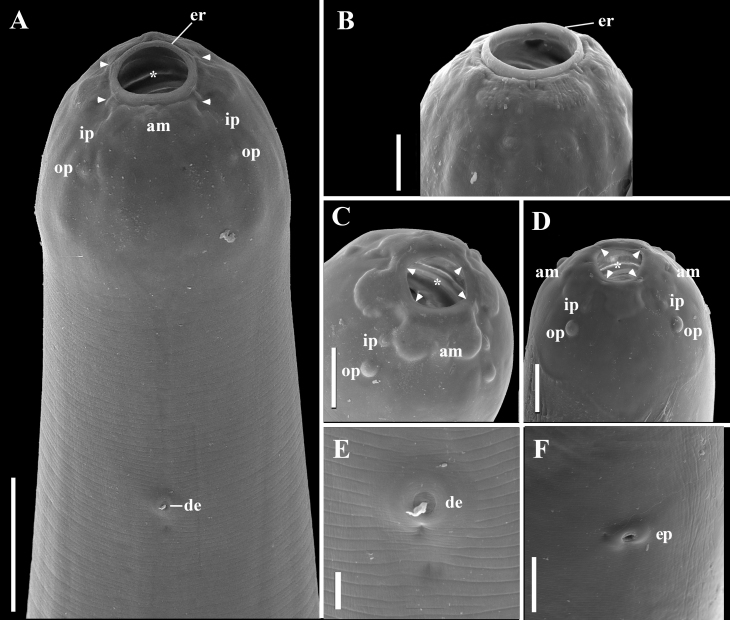
*Spirocamallanus rarus* parasitizing *Propimelodus eigenmanni* by SEM*.* (A) Female, anterior end, lateral view, showing mouth opening (asterisks), with a thick cuticular external ring (er) at the anterior end of the buccal capsule, surrounded by inner circle with four small pore (arrowheads) at base proximal to mouth opening, six elevations, one ventral, one dorsal, and four lateral, and eight papillae distributed in two circles, four internal smaller (ip) and four outer larger (op) and a pair of small lateral amphids (am) and deirid (de). (B) Female, detail of cephalic region, showing mouth opening surrounding by a thick cuticular external ring (er) at the anterior end of the buccal capsule. (C–D) Males, cephalic end, (C) lateral view and (D) dorsal view, both showing circular mouth opening (asterisks), surrounded by inner circle with four small pore (arrowheads) at base proximal to mouth opening, six elevations, one ventral, one dorsal, and four lateral, and eight papillae distributed in two circles, four internal smaller (ip) and four outer larger (op) and a pair of small lateral amphids (am). (E) Female, lateral view, detail of deirid (de). (F) Female, ventral view, detail of excretory pore (ep). Scale bars: A = 50 μm, B–D = 20 μm, E = 5 μm and F = 10 μm.

**Figure 4:**
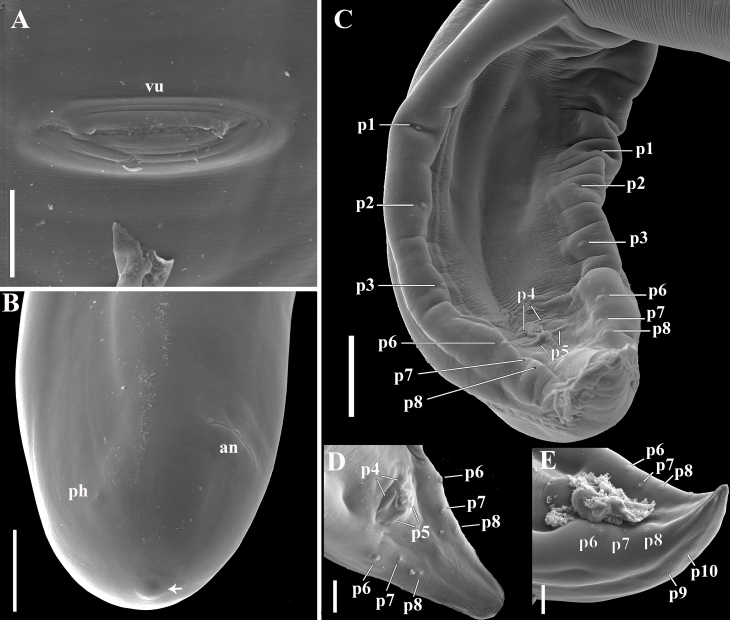
*Spirocamallanus rarus* parasitizing *Propimelodus eigenmanni* by SEM*.* (A) Female, ventral view, detail of vulva (vu). (B) Female, tail, ventrolateral view, showing anus (an), phasmid (ph), and small terminal protuberance (arrow). (C–E) Males, posterior end, (C–D) ventral view, and (E) ventrolateral view, showing caudal papillae. The caudal papillae, p1–p3 precloacal, p4–p5 adcloacal, p6–p10 postcloacal pairs (p9–p10 the phasmids). Scale bars: A = 20 μm, B–C = 50 μm, D - E = 20 μm.

General description, based on 28 specimens observed by optical and 10 specimens observed by scanning electron microscopy: Medium-sized reddish and slender nematodes with delicate and finely transversely striated cuticle. Mouth opening circular, surrounded by inner circle with four small pores at its base proximal (Figure 1C, 3A), six elevations (one ventral, one dorsal, and four lateral), and eight papillae distributed in two circlets (four internal smaller and four outer larger), a pair of small lateral amphids, with a thick cuticular external ring at the anterior end of the buccal capsule, conspicuous in females and inconspicuous in males (Figure 3B, C, D). Buccal capsule, orange-brown, longer than wide, thick walled, barrel-shaped, capsule internally equal between sexes with four serrated spiral ridges, with a well-developed basal ring, armed with six sclerotized tooth–like structures (three large teeth and three smaller teeth) (Figures 1B, 2A, B). Muscular esophagus somewhat shorter than glandular (Figure 1A). Deirids between the buccal capsule (Figure 3E) and a nerve ring, excretory pore immediately below to nerve ring (Figures 2A, 3F). Females are ovoviviparous.

Male description, based on 12 specimens observed by optical and 5 by scanning electron microscopy: Body 12 mm (10–13 mm) long; maximum width at esophageal/intestinal junction 172 (140–207). Buccal capsule including basal ring 93 (83–103) × 73 (67–80); basal ring 5 (3–8). Length/width ratio of buccal capsule 1:0.8 (1:0.7–0.9). Deirids, nerve ring and excretory pore at 147 (128–183), 248 (197–303) and 394 (323–493), respectively, from anterior extremity. Muscular portion of esophagus 643 (433–727) long and 62 (53–76) wide; glandular portion of esophagus 1.034 (827–1.267) long and 75 (53–100) wide. Length ratio of muscular: glandular esophagus 1:0.6 (1:0.5–0.8). Length of entire esophagus and buccal capsule constituting 15% (13–16%) of body length. Posterior end of body ventrally curved. Caudal alae, supported by pedunculate papillae. Ten pairs of caudal papillae: three pairs subventral pedunculate precloacal papillae, equidistant from each other, third pair slightly anterior to cloaca; two pairs ventral transversely elongated sessile adcloacal papillae; and five pairs postcloacal papillae (three pairs subventral pedunculate and two pairs lateral, as with the phasmids) (Figures 1F, G, 2F, 3C, D, E). The first pair of phasmids located immediately below the last pair subventral pedunculate papillae and the second pair of phasmids near the tip of the tail. Spicules dissimilar in form and size, characteristic terminal bifurcation in the larger spicule 583 (533–630) long, shorter spicule fine and lacking bifurcation 205 (143–253) long (Figures 1F, 2 E). Gubernaculum absent. Length of tail 135 (90–173).

Females with larvae and eggs (based on 13 specimens observed by optical and 5 by scanning electron microscopy): 20 mm (15–24 mm) long; maximum width at esophageal/intestinal junction 256 (200–340). Buccal capsule, with four spiral ridges, including basal ring, 112 (102–128) long and 87 (80–112) wide. Basal ring 6 (4–7). Maximum width/length ratio of buccal capsule 1:0.8 (1:0.7–0.9). Deirids, nerve ring and excretory pore 182 (148–220), 289 (237–317) and 434 (300–503), respectively, from anterior extremity. Muscular portion of esophagus 840 (700–967) long and 81 (73–93) wide; glandular portion of esophagus 1.464 (1.153–1.813) mm long and 105 (80–120) wide. Length ratio of muscular: glandular esophagus 1:0.6 (0.6–0.6). Length of entire esophagus and buccal capsule constituting 12% (10–15%) of body length. Vulva pre-equatorial with lips not elevated, 9 (6–11) mm from anterior extremity, 44% of body length (Figures 1E, 2C, 3A). Uterus filled with larvae 390 (323–433) long (based on 20 larvae) and eggs 34 (24–42) long by 29 (22–37) wide (based on 30 eggs). Small lateral phasmid pair. Tail broad, 107 (67–133) long, slightly narrowed near tip, with outlined small terminal protuberance 6 (3–8) (Figures 1D, 2 D, 3B).

Females with eggs (based on 3 specimens by light microscopy): Body 16 mm (15–16 mm) long; maximum width at esophageal/intestinal junction 198 (153–220). Buccal capsule including basal ring, 102 (95–112) × 77 (67–87); basal ring 4 (3–6), four spiral ridges. Maximum width/length ratio of buccal capsule 1:0.8 (1:0.7–0.8). Deirids, nerve ring and excretory pore 167 (150–187), 277 (267–283) and 414 (407–423), respectively, from anterior extremity. Muscular portion of esophagus 815 (760–913) long and 67 (67–67) wide; glandular portion of esophagus 1.173mm (1.127–1.253mm) long and 78 (67–87) wide. Length ratio of muscular: glandular esophagus 1:0.7 (0.6–0.8). Length of entire esophagus and buccal capsule constituting 13% (13–14%) of body length. Vulva pre-equatorial with lips not elevated, 7 (7–7) mm from anterior extremity, 44% of body length. Uterus filled with eggs 27 (20–33) long by 22 (17–27) wide (based on 30 eggs). Small lateral phasmid pair between the anus and the tail tip. Tail broad, 77 (65–92) long, slightly narrowed near tip, with outlined small terminal protuberance 5 (3–7).

Locality: Parauaú River, Breves municipality, Pará state, Brazil.

Parasitic indices: prevalence 36%, mean intensity 6.22, mean abundance 2.24, and range of infection of 1–6 nematodes per fish.

Deposited specimens: voucher specimens were deposited as wet material in the MPEG, Belém, Pará state, Brazil: MPEG.NEM 000432 (males); MPEG.NEM 000433 (females).

After reevaluation of the holotype and representative specimens of *S. rarus* used on the original description and on the redescriptions deposited at CHIOC some morphological observations were added (Figures 5, 6).

**Figure 5:**
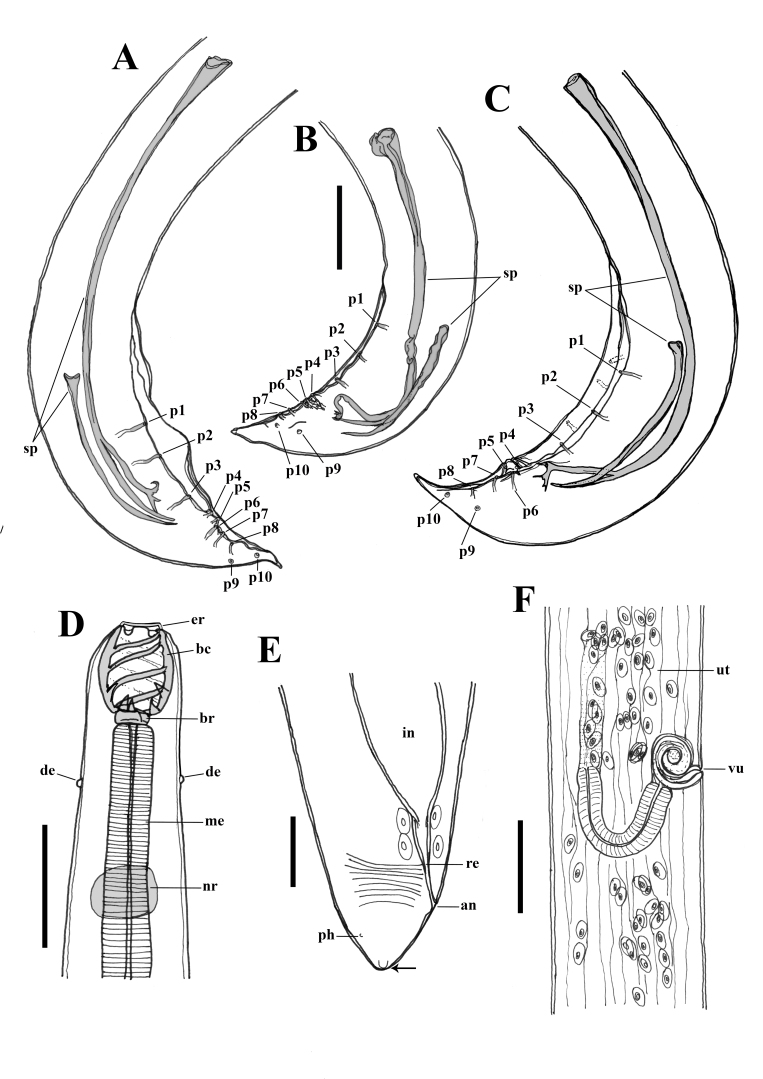
*Spirocamallanus rarus*specimens deposited in the Coleção Helmintológia do Instituto Oswaldo Cruz (CHIOC). (A–C) Males (A = CHIOC 31028b, B = CHIOC 31029 holotype and C = CHIOC 31027a), posterior end, lateral view, showing caudal papillae, p1–p3 precloacal, p4–p5 adcloacal, p6–p10 postcloacal pairs (p9–p10 the phasmids) and spicules (sp). (D) Female (CHIOC 31026b), anterior end, dorsal view, showing external ring (er), buccal capsule (bc), teeth at base of buccal capsule, basal ring (br), muscular esophagus (me), nerve ring (nr) and deirids (de). (E) Female (CHIOC 31027b), posterior end, dorsolateral view, showing intestine (in), rectum (re), anus (an), phasmid (ph) and the tail tip with the button shaped cuticular projection (arrow). (F) Female (CHIOC 31026a), detail of vulvar region, lateral view, showing uterus (ut) and vulva (vu). Scale bars: A–C = 0.1 mm, D = 0.1 mm, E = 0.1 mm and F = 0.2 mm.

**Figure 6:**
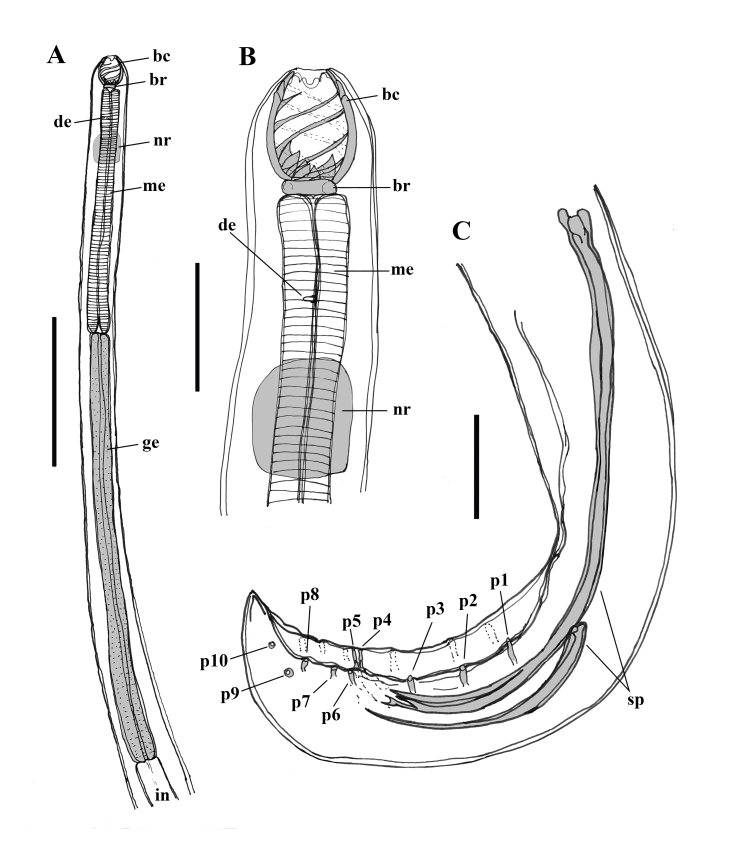
*Spirocamallanus rarus* male, representative specimen deposited in the Coleção Helmintológia do Instituto Oswaldo Cruz (CHIOC 35717). (A) Anterior end, lateral view, showing buccal capsule (bc), basal ring (br), deirid (de), muscular esophagus (me), glandular esophagus (ge), nerve ring (nr) and intestine (in). (B) Anterior end, lateral view, details of buccal capsule (bc), basal ring (br), teeth at the base of buccal capsule, muscular esophagus (me), nerve-ring (nr) and deirid (de). (C) Posterior end, lateral view, showing caudal papillae, p1–p3 precloacal, p4–p5 adcloacal, p6–p10 postcloacal pairs (p9–p10 the phasmids), and spicules (sp). Scale bars: A = 0.4 mm and B–C = 0.1 mm.

The holotype (CHIOC 31029) is an intact male specimen mounted in Canada balsam. Buccal capsule with four ridges and a basal ring with six teeth. Caudal alae supported by pedunculate papillae. Ten pairs of caudal papillae: three pairs subventral pedunculate precloacal papillae, equidistant from each other, third pair slightly anterior to cloaca; two pairs ventral transversely elongated sessile adcloacal papillae; and five pairs postcloacal papillae (three pairs subventral pedunculate and two pairs lateral, as with the phasmids). First pair of phasmids located immediately below the last pair subventral pedunculate papillae and the second pair of phasmids near the tip of the tail.

Voucher CHIOC 31026a, an intact female specimen, except for the buccal capsule that is ruptured at its anterior end, is a total mount in Canada balsam. Buccal capsule with four ridges, a basal ring with six teeth, and a thick cuticular external ring conspicuous at its anterior end. Deirids and excretory pore inconspicuous. Vulva pre-equatorial with lips not elevated. Uterus filled with eggs. Small lateral phasmid pair between the anus and the tail tip. Tail broad, slightly narrowed near tip, with outlined small terminal protuberance.

Voucher CHIOC 31026b, a female specimen, with the cuticle ruptured in various parts of the body, from below the junction of the glandular esophagus with the intestine, and the end of the tail is missing, wholemount preserved in Canada balsam. Buccal capsule with four ridges and a basal ring with six teeth, a thick cuticular external ring conspicuous at its anterior end. Deirids conspicuous. Vulva pre-equatorial. Uterus filled with eggs.

Voucher CHIOC 31026c, a male specimen, only the part of the tail with the broken cuticle is mounted, preserved in Canada balsam. Voucher CHIOC 31027a, an intact male specimen, except for buccal capsule that is ruptured at its anterior end, wholemount preserved in Canada balsam. Buccal capsule with four ridges and a basal ring with six teeth. Presented the same number and distribution of caudal papillae (including the phasmids) as in the specimens collected in this study.

Voucher CHIOC 31027b, an intact female specimen, wholemount preserved in Canada balsam. Buccal capsule with four ridges, a basal ring with six teeth, and a thick cuticular external ring conspicuous at its anterior end. Vulva pre-equatorial with lips not elevated. Uterus filled with eggs. Small lateral phasmid pair between the anus and the tail tip. Tail broad, slightly narrowed near tip, with outlined small terminal protuberance.

Voucher CHIOC 31028a, a male specimen, with anterior and posterior regions broken, preserved in Canada balsam. Buccal capsule, broken, with four ridges. Only postcloacal papillae visible, precloacal papillae and phasmids inconspicuous.

Voucher CHIOC 31028b, a male intact specimen, except for buccal capsule that is ruptured at its anterior end, wholemount preserved in Canada balsam. Buccal capsule with four ridges. Presented the same number and distribution of caudal papillae (including the phasmids), as in the specimens collected in this study.

Voucher CHIOC 31028c, a female intact specimen, except for buccal capsule that is ruptured at its anterior end, wholemount preserved in Canada balsam. Buccal capsule with four ridges, a basal ring with six teeth, and a thick cuticular external ring conspicuous at its anterior end. Vulva pre-equatorial with lips not elevated. Uterus filled with eggs. Small lateral phasmid pair between the anus and the tail tip. Tail broad, slightly narrowed near tip, with outlined small terminal protuberance.

Voucher 35717, a male intact specimen, wet material. Buccal capsule with six teeth at its base. Deirids between the buccal capsule and the nerve ring. The glandular esophagus is the larger, measuring 1,280 µm long and not 280 µm long, as it had been described. Presented the same number and distribution of caudal papillae (including the phasmids), as in the specimens collected in this study.

## Remarks

The nematodes recovered from the intestine of *P. eigenmanni* catfish from the Parauaú River were morphologically and morphometrically analyzed and taxonomically determined as *S. rarus* according to the description and redescriptions of the species (Travassos et al., 1928; Pinto et al., 1974; Melo et al., 2012). In this study the specimens of *S. rarus* were morphologically characterized by having a buccal capsule with four internal spirals, a basal ring armed with six teeth (three large and three small teeth), 10 pairs of pedunculated papillae on the caudal wing, three being elongate subventral precloacal pairs, two sessile ventral adcloacal pairs, and five pairs postcloacal (three elongate subventral pairs, and two additional sessile lateral pairs, as phasmids) and unequal spicules, with the larger spicule bifurcated in males, this characteristic spicules is crucial for this species determination. Although some *Spirocamallanus* species present a small number of spirals in the buccal capsule, such as *S. amarali*, *S. iheringi*, *S. pexatus* (= *S. chimusensis* Freitas & Ibáñez, 1968) 6–8, 3–9, 3–7, respectively, only *S. rarus* has four (4) internal spirals in the buccal capsule (Pinto & Noronha, 1976; Moravec, 1998; Moravec et al., 2004a), and the unequal spicules, with the larger spicule bifurcated (Pinto et al., 1974; Melo et al., 2012).

In this study we used a large number of specimens collected and this fact allowed us to increase the morphometric range for all the morphological structures (Table 1). Beyond the morphological characterization and morphometry of the spicules, some new data were observed and added in relation to this distribution of the caudal papillae, which have been considered in the original description and in subsequent redescriptions as being four precloacal pairs and four postcloacal pairs (Travassos et al., 1928; Pinto et al., 1974; Melo et al., 2012). Furthermore, detailed study of the specimens collected in this study using light microscopy and SEM, allowed us to unveil the internal structures and topography, adding to the description, details about the striated cuticle, cephalic papillae, deirids, excretory pore, vulva, phasmids, and anus of the females. Some of these structures also had their morphological characteristics confirmed through reevaluation using optical microscopy of the holotype and representative specimens deposited at CHIOC.

**Table 1:** Morphological and morphometric data of *Spirocamallanus rarus* specimens collected on the present study and on the previous references in Brazil.

**Hosts and localities**	*Spirocamallanus rarus* **present study**			*S. rarus* Travassos et al. (1928)	*S. rarus* Pinto et al*.* (1974)		*S. rarus* Melo et al. (2012)
	*Propimelodus eigenmanni* **Breves, Pará State**			*Pimelodella lateristriga* **Pirassununga, São Paulo State**	**catfish unidentified Maicuru, Pará State**		*Satanoperca jurupari* **Belém, Pará State**
	**Males**	**Females** *	**Females** **	**Male**	**Males**	**Females**	**Male**
Body (L) mm	10–13	15–24	15–16	5.3	9.2–13.5	11.1–25.6	11.3
Body (W)	140–207	200–340	153–220	110	180–200	200–340	220
Buccal capsule (L)	83–103	102–128	95–112	72	84–100	84–180	100
Buccal capsule (W)	67–80	80–112	67–87	56	–	46–110	80
Ratio buccal capsule (W/L)	1:0.7–0.9	1:0.7–0.9	1:0.7–0.8	1:0.8	–	–	0.8
Number of spiral ridges	4	4	4	3–4	3–4	3–4	4
Theeth	Present	Present	Present	Present	Present	Present	Present
Deirid^a^	128–183	148–220	150–187	–	–	–	–
Nerve ring^a^	197–303	237–317	267–283	250	160–210	220–230	290
Excretory pore^a^	323–493	300–503	407–423	380	–	–	437
Muscular esophagus (L)	433–727	700–967	760–913	680	500–810	650–1390	740
Glandular esophagus (L)	827–1.267	1.153–1.813	1.127–1.253	640	800	660–1640	280
Ratio Me/Ge (L)^b^	1:0.6	1:0.6	1:0.7	1:1.1	1:0.8	1:0.9	0.4:1
Vulva^a^ mm	–	6–11	7–7	–	–	–	–
Precloacal papillae (pairs)	3	–	–	4	4	–	4
Adcloacal (pairs)	2	–	–	–	–	–	–
Postcloacal papillae (pairs)	5	–	–	4	4	–	4
Small spicule (L)	143–253	–	–	170	220–280	–	212
Large spicule (L)	533–630	–	–	400	630	–	585
Tail^b^	90–173	67–133	65–92	120	100	150	112

L= length, W = width.

* females with eggs and larvae;

** females with eggs only; measurements are in micrometers unless otherwise indicated;

a distance from anterior extremity; ^b^ distance from posterior extremity;

b Ratio Me/Ge (L) = Ratio between the lengths of the muscular esophagus and glandular esophagus.

In the original description, Travassos et al. (1928) provided measurements of a single specimen collected from *Pimelodella lateristriga* (Lichtenstein, 1823) from Emas, Pirassununga, São Paulo state. Pinto et al. (1974) redescribed the holotype and more data were added based on specimens collected from a catfish from the Amazon River, Maicuru, Pará state, where the shape and size of the two spicules were detailed, especially the larger with the characteristic bifurcate spicule. Morphological data from collected females were also added to this description. Melo et al. (2012) reported one unique specimen recovered from *S. jurupari*, from Guamá River, Belém, Pará state, and made the identification of the species based on the largest spicule characteristic and the eight pairs of caudal papillae (four precloacals and four postcloacals) and also described the glandular esophagus as smaller than the muscular esophagus. Morphometric comparisons between the existing descriptions based on the references (op. cit.) of *S. rarus* and the additional data observed in this study (Table 1) made it possible to show some differences, mainly in the number of caudal papillae.

In this paper, after morphologic and morphometric reevaluation using through light microscopy, the specimens collected by Travassos et al. (1928), Pinto et al. (1974) and Melo et al. (2012) deposited in the CHIOC, under the numbers, 31.029 (holotype, male); 31.026 a-b (females), c (male), 31.027 a (male), b (female), 31.028 a-b (males), c (female), and 35717 (male), respectively, it was observed that these specimens also presented the same number and distribution of caudal papillae (including the phasmids) in males as in the specimens collected in this study. In the Melo et al. (2012) specimen was possible to observe the deirids, and the glandular esophagus was larger than previously described, with a length of 1.280 µm and not 280 µm, as, allowing this detail to be corrected.

In this study drawings were added to the morphological data based on observations of the holotype and representative specimens deposited at CHIOC (Figures 5 and 6), such as the conspicuous anterior chitinous ring in the mouth of females but inconspicuous in males; the basal ring of buccal capsule armed with 6 teeth, and the presence of deirids and phasmids in males and females, which had not been observed in these previous articles.

## Discussion

Since the original description of *S. rarus* parasitizing *P. lateristriga* (Siluriformes: Heptapteridae) and *Rhinodoras dorbignyi* (Kner, 1855) (Siluriformes: Doradidae) in Emas, Pirassununga, São Paulo (Travassos et al., 1928) that was based on only one whole male, and on another damaged and un-deposited specimen parasitizing *R. dorbignyi*, different researchers (Pinto et al., 1974; Moravec, 1998; Giese, 2010; Melo et al., 2012) have recorded the presence of this parasite in new hosts, such as *Pimelodus maculatus* Lacepède, 1803, *P. albicans* (Valenciennes, 1840), *Synodontis clarias* (Linnaeus, 1758) and an unidentified catfish (all Siluriformes) and *Serrasalmus* sp. (Cypriniformes: Serrasalmidae). This has expanded the geographic distribution of this nematode species to Argentina, Paraguay and Peru.

Previous reports of the nematode *Spirocamallanus rarus* in the the northern region of Brazil were made by Pinto et al. (1974), parasitizing an undetermined catfish, in the Amazon River, Maicuru, and by Giese (2010) and Melo et al. (2012) reporting *S. rarus* parasitizing the fish *Ageneiosus ucayalensis* Castelnau, 1855 (Siluriformes: Auchenipteridae) and *Satanoperca jurupari* (Heckel, 1840) (Cichliformes: Cichlidae), in Pará state, respectively. There are also reports by Negreiros et al. (2018) and Cavalcante et al. (2020) of this species parasitizing the intestine of *Pimelodus blochii* Valenciennes, 1840 (Siluriformes: Pimelodidae) collected from the Acre, Iaco, Xapuri Rivers in Acre state, respectively. Therefore, ours is the first reported occurrence of this species parasitizing *P. eigenmanni*.

Almost 100 years after the description of *S. rarus* (Travassos et al., 1928), in this paper we present the first data observed by scanning electron microscopy. In this study the observations obtained by light and scanning electron microscopy, clarified and added many morphological aspects of this species and some were observed for the first time, on the cephalic structures, such as the papillae distributed in two circlets and amphids, the deirids and excretory pore locations on the anterior region, the topography of the cuticle, the number and location of caudal papillae in males, the vulva and phasmids on the tail of females.

Worldwide, different studies have presented data from SEM and added important information for morphological characterization of Procamallaninae with proposal of new species and redescriptions over at least the last 20 years (González-Solís et al., 2002; Moravec et al., 2003, 2004a, b, 2006; Giese et al., 2009; Moravec & Justine, 2011; Moravec & Jirků, 2015; Ailán-Choke et al., 2018, 2019; Rivadeneyra et al., 2020) reinforcing the importance of this technique for the characterization of these nematodes.

## Conclusion

This study reports the occurrence of the *S. rarus* parasitizing *P. eigenmanni* in the Pará state, adding data from light microscopy and scanning electron microscopy, and a re-examinatiuon of specimens of this species deposited at CHIOC. This expands the knowledge about this species and contributes to a better understanding of the morphology of camallanid nematodes parasitizing fishes in Brazil.

## Data Availability

Data will be made available on request.
